# Where to From Here?

**DOI:** 10.3389/fmolb.2022.848444

**Published:** 2022-03-25

**Authors:** Robert Schleif, Manuel Espinosa

**Affiliations:** ^1^ Department of Biology, Johns Hopkins University, Baltimore, MA, United States; ^2^ Department of Molecular and Cell Biology, Centro de Investigaciones Biológicas Margarita Salas, CSIC, Madrid, Spain

**Keywords:** artificial intelligence, biocomputing, deep learning, protein structure and function, prediction of protein structures

## Abstract

The biological-biochemical community has been shocked and delighted by the remarkable progress that has recently been made on a problem that has consumed the attention, energy, and resources of many, if not most of the scientists in the field for the past 50 years. The problem has been to predict the tertiary structure of a protein merely from its amino acid sequence. Nature does it easily enough, but it has been an incredibly difficult problem, often considered intractable, for humankind. The breakthrough has come in the form of two computer-based approaches, AlphaFold2 and RoseTTAFold in conjunction with factors such as the use of vast computing power, the field of artificial intelligence, and the existence of huge protein sequence databases. The advancement of these tools depended upon and was stimulated by the last 50 years of development of smaller and smaller and more and more powerful electronics components, mainly processors and memory. Along with the problem of protein folding, determining the function or mechanism of action of proteins has similarly limped along as did protein folding until the recent breakthroughs. Perhaps AlphaFold2 and RoseTTAFold can substantially aid in protein mechanistic studies. Now it is not completely insane to consider what might be the next grand challenge in biochemistry-biology. We offer several possibilities.

## 1 Introduction

### 1.1 What Just Happened

Easily stated and understood statements often possess unusual powers of attraction. Hence, for more than half a century, the objective of predicting the tertiary structure of amino acid sequences has occupied the position as the most attractive, most researched, and most interesting problem in Biology and Biophysics. It is not just the easily understood and articulated objective of predicting structure that has given it such a prominent position. Another widely quoted and easily understood dictum explains this interest as deriving from “Structure determines function.” Behind our interest in structure lies our deeper interest, sometimes explicitly stated, but often implicitly implied. That is, our desire to be able to manipulate and control our environment and destiny. With respect to Medicine and Biology this means to be able to design drugs and to be able to design and build proteins that possess reasonable enzymatic or structural properties. Knowing the structure of existing proteins and being able to accurately predict the structures of hypothetical proteins has therefore been the major driving force for learning and predicting the structures of proteins. The drive to determine protein structure led to the development of X-ray determination first of myoglobin by Kendrew and others (Kendrew et al., 1958) and ultimately to the 185,541 Structures (as of 3 January 2022) deposited in the Protein Data Bank, PDB (https:www.rcsb.org), which have been laboriously determined by many hundreds of researchers over the past half century.

In theory, structure could also be predicted rather than determined experimentally. Molecular modelling of proteins with no or little similarity to existing structures (the so-called *ab initio* modelling) is a most demanding objective in tertiary structure prediction. Thus, at the beginning of the XXI century, the Structural Genomics initiative proposed a large scale effort for the determination of protein structures, irrespective of whether their function was known (Mariani 2004). An example of a project where an attempt was made to determine structure and function, is provided by a protein termed “Putative Mga family transcriptional regulator from *Enterococcus faecalis*” (PDB 3SQN), deposited by Osipiuk et al., in 2011 (Osipiuk, J.; Wu, R.; Jedrzejczak, R.; Moy, S.; Joachimiak, A., to be published). The protein is encoded by the enterococcal EF3013 gene and its potential role in transcriptional control was not determined until 5 years later, when it was demonstrated to act as a global transcriptional activator (MafR) of numerous enterococcal genes (Ruiz-Cruz et al., 2016).

The determination of protein structure by X-ray diffraction has been augmented by Nuclear Magnetic Resonance (NMR), and more recently by Cryo-Electron Microscopy (EM). Both structure determination methods are difficult, lengthy, require very expensive instruments, and are successful in determining a protein’s structure only part of the time. Given the difficulty of determining protein structures, there has been considerable and growing interest over the past half century in predicting protein structure.

Over the past 26 years, prediction of protein structure has been enormously stimulated by the biannual competition, Critical Assessment of protein Structure Prediction (CASP) organized by the Protein Structure Prediction Center (https://predictioncenter.org/). Until fairly recently, progress as measured in the CASP1 competitions and meetings was incremental at best. The prediction capabilities for proteins up to about 110 amino acids slowly rose, reaching an accuracy when compared to experimentally determined structure of about an Angstrom for perhaps 70% of proteins attempted (Moult et al., 2011; Moult et al., 2014).

Beginning with CASP12 there was a dramatic increase in the accuracy of predictions (Senior et al., 2020). The use of huge databases of protein sequences (big data) as well as the use of advanced deep learning artificial intelligence (AI) techniques were primarily responsible for this advance (Kryshtafovych et al., 2019). These permitted testing and then utilizing the obvious idea that two amino acid changes in a protein, one which impairs activity and a second which restores activity, result from amino acids that very likely contact one another or lie very close to one another in the tertiary structure of a protein. Such covarying amino acid pairs are found in evolutionarily related proteins, that is, in a protein’s homologs found in the sequence databases. Application of structural constraints based on covarying amino acids dramatically increased both the size of proteins that could be predicted, and the quality of the predictions. Nonetheless, prediction of protein structure remained uncertain and of variable quality.

In light of the very long and slow progress in both experimental determination and computational prediction of protein structure, it was therefore both a shock and a delight to hear of the remarkable advances achieved in CASP13 and CASP14, where the AI approaches were described first for the AlphaFold program developed by group of scientists in the Deep Mind company, with the support of Google and backed by an enormous amount of computational power (Senior et al., 2020). The first iteration of AlphaFold was based on a neural network that predicted the distance between parts of a target protein. Next we learnt of the tr (transform restrained) RoseTTA program developed by the laboratory of David Backer (Anishchenko et al., 2021), later implemented into a server (Du et al., 2021). The usefulness and accuracy of trRoseTTA was soon demonstrated for the pneumococcal sigma factor, SigA, protein compared to the already solved *Escherichia coli* counterpart (Solano-Collado et al., 2021). Another interesting approach, also based on neural networks and termed Recurrent Geometric Networks, was published and claimed to be faster than the AlfaFold program although it may be of less accuracy (AlQuraishi 2019). These AI-based programs fairly routinely predict the structures of new proteins to unprecedented accuracy (Berg 2021).

After the first giant steps forward, there was the second version of AlphaFold named, unsurprisingly AlphaFold2. The development of the advanced deep-learning AlphaFold2 method (CASP14) allowed the construction of three-dimensional models with an accuracy equal to or superior to the experimental accuracy (GDT_TS > 90) for about 70% of the targets, and of a very high accuracy for almost 90% of the targets. By mid-2021, came the release of the basics of the network code and the prediction for nearly 250,000 structures from several model organisms together with the European Molecular Biology Laboratory’s European Bioinformatics Institute (EMBL-EBI) in Hinxton, United Kingdom (Jumper et al., 2021). Not surprisingly, John Jumper who was the first author, was included in the Nature’s list of ten scientists achieving key developments in 2021 (https://www.nature.com/immersive/d41586-021-03621-0/index.html?utm_source=Nature+Briefing&utm_campaign=f5ce3c484a-briefing-dy-20211216&utm_medium=email&utm_term=0_c9dfd39373-f5ce3c484a-46322698#section-7cgEBpkV9L).

Finally, and included within the breakthrough articles of the journal Science for 2021, there was the article by the Baker and Cong laboratories in which a version of the rapidly computable RoseTTAFold was combined with the AlphaFold programs (Humphreys et al., 2021). The approach used co-evolutional protein-protein interactions to study 8.3 million pairs of yeast proteins. A total of 106 previously unidentified assemblies and 806 that were structurally uncharacterized was the result of this powerful approach, extending the range of deep learning based structural protein modelling. The Baker laboratory has implemented the very busy Robetta on-line server (https://robetta.bakerlab.org/), based on RoseTTAFold, that allows the unexperienced user to obtain predicted three-dimensional models of any given protein starting from the amino acid sequence of a protein.

At the time that this manuscript was being handled by the Editorial Office, the online access to the AlphaFold2 Database, linked to the EMBL-EBI website (https://alphafold.ebi.ac.uk/) was accessible (Varadi et al., 2022). The output has an impressive predicted structure in milliseconds with the (in our opinion) drawback of not being yet linked to the NCBI database. Nearly at the same time (January, 11th, 2022), the journal Nature Methods published an editorial and two adjoining papers declaring the Protein Structure Prediction as the Method of the Year 2021 (Editorial 2022).

## 2 Where We Now Stand

**FIGURE 1:**
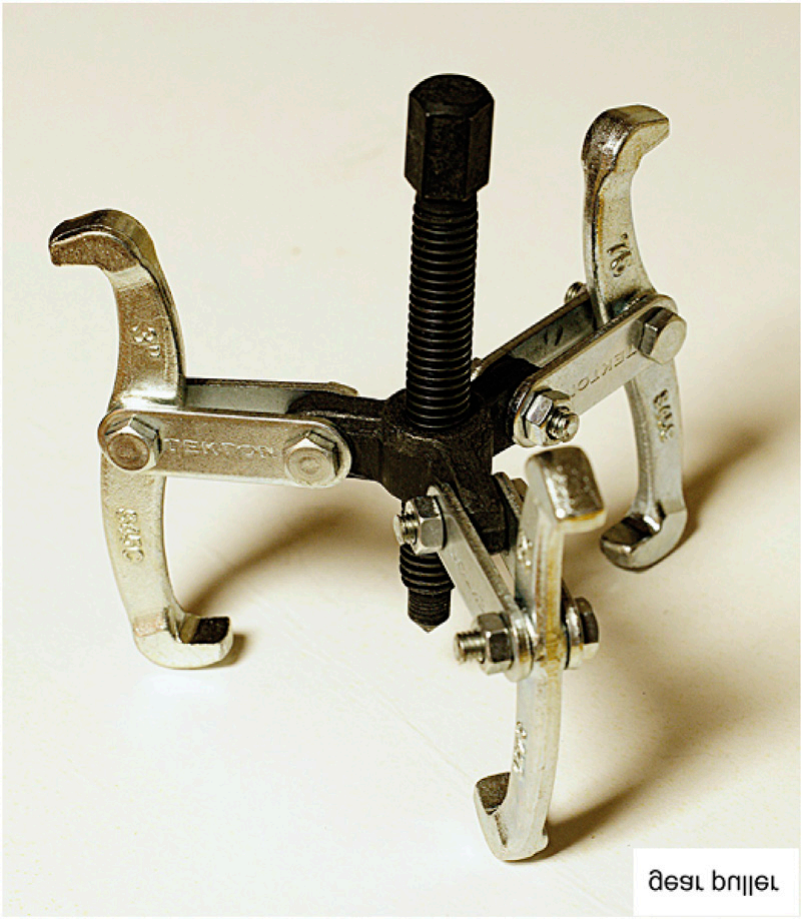
Image of a tool whose function is not obvious from its structure. Its name and function are given in the bottom right corner as a horizontal reflection.

Without a doubt, we cannot fully understand the mechanism of action of a protein without knowledge of its structure. On the other hand, and up to now, knowledge of the structure of a protein has only rarely told us how the protein works. That is to say that while the structures of many proteins (alone or complexed with their targets) have been solved, in the great majority of the cases, examination of the structure alone has proved to be insufficient to reveal the critical details of the proteins’ mechanism of action. A clear example is provided by some instruments or gadgets that, even though we can see and analyze them, most of us cannot provide an explanation for what they do. Figure 1 shows an example of such a gadget. It is worth pointing out the distinction between possessing a long list of facts about the behaviour of a protein and actually possessing an understanding of the protein’s action.

One way of characterizing our current level of understanding protein structure and functions is to compare “external” and “internal” events. Currently used technology allows us to learn a considerable amount what other proteins a particular protein interacts with, the strengths of these interactions, and sometimes the physiological consequences of damaging these interactions. However, we know much less about the “internal” events in protein function. Not only must proteins fold, and refold if they have suffered a fluctuation and partially unfolded, but they usually must bind one or more small molecule ligands or macromolecular targets. Proteins must do this with high specificity and with kinetics appropriate to the time scales dictated by intracellular conditions and cell growth. Finally, a good many of proteins must also undergo a conformational change in response to binding a target molecule and in the altered conformation, must possess an altered binding activity for another molecule. This phenomenon is generally referred to as an allosteric change, and allosterism is a key component not only in gene regulatory proteins, but also in proteins that control the flow of metabolites down biosynthetic and degradation, or perhaps catabolic and anabolic pathways.

As expected from our relatively high level of “external” knowledge of proteins, the past 50 years has seen the development of many powerful techniques for the acquisition of such knowledge. Techniques for the study of “internal” protein events are much less well developed, although a number of techniques can be listed, such as structure determination by X-ray crystallography or NMR or Cryo-EM followed by computational approaches, molecular dynamics, fluorescence studies, etc. While these techniques can yield much information, their application has required specialized training and experience that is not available to every laboratory interested in a given protein or family of proteins. Conversely, structuralists may lack of interest or skills required to use their techniques to learn much about protein function.

Another way of describing the bottlenecks to advancement is that the development of Molecular Biology allowed physiological observations made on populations of cells to be explained in terms of molecules within the cell. That is, understanding has been brought to the molecular level by determining what macromolecules exist and what they do. Unfortunately, progress in understanding by working from the top down has slowed.

It is also possible to work from the bottom up. Quantum mechanics determines chemistry, which in turn determines biochemistry. Alas, an impasse is also reached in this approach. Specifically, we can predict from basic principles the structures and properties of molecules containing perhaps as many as 25 atoms, but biological macromolecules contain tens of thousands of atoms. Thus, the “bottom up” approach cannot tell us, up to now, about internal events in proteins, and hence cannot yet aid us in understanding or in engineering proteins. Ultimately, of course, the top down and bottom up approaches must fuse. Likely the fusion will be at the level of the action of bio-macromolecules. This should provide us with a complete understanding of biological phenomena at the molecular level as well as the ability to design macromolecules that will perform desired and valuable functions. Upon the fusion, biology will cease being an archeological or geological exploration and instead, will become more of an engineering discipline.

## 3 Where Next

After these recent advances, we could ask whether the experimental protein structure determination industry thus been rendered obsolete with a couple of strokes? We think that the answer is “Largely yes”. Of course, one will still seek verification of predicted structure. And, of course, we would like to replace black box prediction schemes with deeper understandings of the physical laws that underlie the ability of a polypeptide chain to fold into a defined tertiary structure. Thus we agree that the prediction programs must be further developed (Tong et al., 2021), and some proposals have been made on how to proceed beyond AlphaFold2 (Bagdonas et al., 2021; Buel and Walters 2022).

Whatever the further developments, what next? Much yet remains in the area of prediction, for example, predicting structure for unusual environments or temperatures. Still, the thrust of research on proteins should, and eventually will, shift to determinations of mechanisms and the predictions and understandings of the role of protein dynamics and flexibility in the activities of proteins. While it would be nice to be able to design drugs based on the (predicted) structures of target proteins, we are still some distance from this goal, with protein and ligand flexibility one of the obstacles in our way. Another may be insufficiently precise knowledge of the interatomic forces involved in protein-ligand binding. Research will also shift to these important questions.

We are also still very far from being able to discern, predict, understand, or knowledgeably alter the properties of some classes of proteins. For example, the determination of the mechanisms by which their small molecule effectors alter the DNA binding affinity of the bacterial proteins, Lac repressor, cAMP receptor, and AraC have come to be largely, but not completely understood as the result of many hundreds of person years—and this effort was required after the relevant tertiary structures had been determined. It remains to be seen if AlphaFold2 and RoseTTAFold can be used directly to accelerate mechanistic studies of other proteins with complex behaviors. More likely, additional dramatic developments in computational biochemistry will be required.

At this point it is impossible to predict what objective might follow the half-century quest to accurately predict protein structure from amino acid sequence. Several projects come to mind: as stated earlier, to be able to design and build a protein with nearly any desired physically possible properties, to be able to determine all the properties of a protein from its structure (or equivalently, its amino acid sequence), most notably, its biological function, and finally, to be able to design small molecule inhibitors and activators of the biological activity of most proteins found in nature or designed for specific purposes.

## Data Availability

The original contributions presented in the study are included in the article/Supplementary Material, further inquiries can be directed to the corresponding authors.
